# Underwater Highly Pressure-Sensitive Fabric Based on Electric-Induced Alignment of Graphene

**DOI:** 10.3390/ma16041567

**Published:** 2023-02-13

**Authors:** Peiru Zhang, Lili Gu, Weiwei Liu, Dengteng Ge, Lili Yang, Ying Guo, Jianjun Shi

**Affiliations:** 1 State Key Laboratory for Modification of Chemical Fibers and Polymer Materials, College of Materials Science and Engineering, Donghua University, Shanghai 201620, China; 2 Department of Applied Physics, Member of Magnetic Confinement Fusion Research Center, Ministry of Education, College of Science, Donghua University, Shanghai 201620, China; 3 China Construction Advanced Technology Research Institute, China Construction Third Engineering Bureau Group Co., Ltd., Wuhan 430075, China; 4 Institute of Functional Materials, Donghua University, Shanghai 201620, China

**Keywords:** wearable sensor, superhydrophobic, graphene, electric-induced alignment, plasma

## Abstract

Wearable pressure sensors have received widespread attention owing to their potential applications in areas such as medical diagnosis and human–computer interaction. However, current sensors cannot adapt to extreme environments (e.g., wet and underwater) or show moderate sensitivity. Herein, a highly sensitive and superhydrophobic fabric sensor is reported based on graphene/PDMS coating. This wearable sensor exhibits great superhydrophobicity (water contact angle of 153.9°) due to the hydrophobic alkyl long chains and rough structure introduced by the Ar plasma. Owing to the network structure created by the electric-induced alignment of graphene sheets, an enhanced sensitivity (ΔI/I_0_ of 55) and fast response time (~100 ms) are observed. Due to its superhydrophobicity and sensitivity, this wearable sensor demonstrates efficient and stable monitoring of various underwater activities, including pressure, blowing, and tapping. Our approach provides an alternative idea for highly sensitive wearable sensors while broadening the practical application scope.

## 1. Introduction

Wearable pressure sensors [1,2,3,4,5,6,7] have attracted much attention in human surface motion detection, biomedical detection, and electronic skin. Traditionally, there are three categories of pressure sensors: piezoelectric, piezoresistive, and pressure-capacitance. Compared with other types, piezoresistive pressure sensors [8,9,10,11] have unique advantages, such as ease of manufacture and low energy consumption. In general, wearable piezoresistive pressure sensors are obtained by loading coatings on a flexible substrate that converts externally applied pressure or mechanical forces into changes in current or resistance through signals. However, current sensors still suffer from mild sensitivity or a lower service life in extreme environments. For example, for underwater monitoring, the sensor is attacked by water, and the device easily short-circuits, resulting in severe signal distortion in practical applications.

Researchers have introduced superhydrophobic material on the sensor surface to improve its resistance to harsh conditions. For example, Ni et al. [12] established a stable reduced graphene oxide/PPy/poly(dimethylsiloxane) (PDMS) polyurethane (PU) sponge (GPPS) sensor by the in situ reduction of graphene oxide and deposited polypyrrole nanoparticles grown on the sponge surface. Chen et al. [13] successfully prepared a multifunctional flexible nanocomposite coating using multibranched polyurethane and silica nanoparticles modified with graphene. Ni et al. [14] prepared a superhydrophobic conductive cotton fabric by impregnating a PDA/reduced graphene oxide solution with copper particles grown in situ and then modified with stearic acid. Although the above sensors have good superhydrophobicity and detection performance, the sensitivity is moderate and the preparation process is relatively complicated. In addition, only a few studies have explored the effect of the spatial orientation and arrangement of interlayer graphene nanosheets on the sensor’s performance. Notably, the contact area between graphene layers changes after the action of the electric field, which in turn improves composite interlayer properties [15]. This also greatly enhances the durability and cycle performance of nanocomposites. Therefore, the demand for multifunctional polymer nanocomposites is increasing. Furthermore, the graphene nanosheets have a tendency to be oriented and aligned in the polymer matrix under the action of external force, and the obtained nanocomposites have unique piezoresistivity. Kim et al. [16] reported epoxy-graphite nanocomposites which were aligned along the electric field direction under the induced action of an AC electric field. The tensile modulus and strength of these materials were found to be anisotropic. Chen et al. [17] prepared directionally oriented graphite nanosheets using an AC electric field and prepared polyester resin nanocomposites, and found that the conductivity of the composites along the directional orientation was significantly improved. Chen et al. [18] prepared a vertical pressure sensor with a single graphene layer using a plasma chemical vapor deposition method. By introducing an amplification structure into the sensor, the detection capability of the sensor was successfully improved. Fan et al. [19] constructed a novel MXene-based textile pressure sensor by assembling MXene on a hollow structured three-dimensional knitted fabric. The designed textile sensor has several excellent sensing properties, such as high sensitivity, fast response time, good stability (over 20,000 cycles), and excellent waterproof capability.

In this experiment, a simple strategy was used to introduce an electric field into the preparation process of the conductive fabric to obtain a graphene interlayer-amplified structure. When an external force was applied, the additional structural deformation of the graphene sheet increased the sensor’s output. For underwater sensing, the polyester fabric obtained after plasma treatment had excellent superhydrophobicity and could be placed underwater to sense underwater motion (water contact angle (WCA) = 153.9°). New ideas were proposed for the design and development of future underwater intelligent equipment.

## 2. Materials and Methods

### 2.1. Materials

Polyester fabrics (plain knitted, thickness ~1 mm, 120 g/m^2^) were purchased from Miandu Textile Co., Ltd. (Zhejiang, China). All fabrics were washed with Detergent 209 aqueous solution (Analytical Purity, Wangnilai Co., Ltd., Guangzhou, China,). The poly(dimethylsiloxane) precursor (MW770) was purchased from Alfa Aesar Chemical Company. Multilayer graphene (thickness less than 100 nm) was purchased from Kaisa (Guangdong, China) Materials Co., Ltd. Anhydrous ethanol (AR ≥ 99.7%) and argon (99.99%) were purchased from Changzhou Hongsheng Fine Detail Co., Ltd. (Changzhou, China) and Shanghai Haoqi Gas Co., Ltd. (Shanghai, China).

### 2.2. Preparation of Superhydrophobic Fabric Sensor

Preparation of superhydrophobic fabric sensor: the deposition of graphene/PDMS precursor, alignment induced by an electric field, and radio frequency capacitively coupled Ar plasma (RF-CCP) treatment. First, graphene and ethanol solutions were ultrasonically dispersed and exfoliated for 2 h, and the poly(dimethylsiloxane) precursor was then added to ultrasonic and mixed for 1 h (Table S1). The prewashed fabric was dipped in the coating solution for 2 h by ultrasonic impregnation, and the impregnated fabric was then dried and cured in a DC electric field (DC high voltage power supply (SL10P1200 0−10 kV) purchased from Spellman Electronic Technology Ltd., Hauppauge, NY, USA) (Figure S1) for 2 h. The fabrics after the electric field action were plasma-treated in a radio frequency capacitively coupled plasma (RF-CCP) (Figure S2) reactor (AP-600, North-March, USA) to obtain superhydrophobic conductive polyester fabrics. The fabric pressure sensor was made of a stack of six superhydrophobic conductive fabrics. Both ends of the sensor are encapsulated with conductive copper tape to reduce the effect of contact resistance during testing. The sensor obtains the most stable electrical signal output.

### 2.3. Characterization

The surface morphology of the polyester fabrics was tested using a field emission electron microscope (Hitachi S-4800, Hitachi, Japan). Water contact and rolling angles were measured using a professional A-200 instrument (Ningbo Haimeishi Testing Technology Co., Ltd., Ningbo, China). The test system consists of an electrochemical workstation (CHI660E), a sensor, and a lab-built applied pressure device (Table S2). An abrasion resistance test was performed by a commercial crock meter (Y571N, Nantong Hongda Instrument Company, Nantong, China) under 15.6 kPa (ISO 105-X12:2001) based on the AATCC 8-2007 colorfastness method.

## 3. Results and Discussion

**Figure 1:**
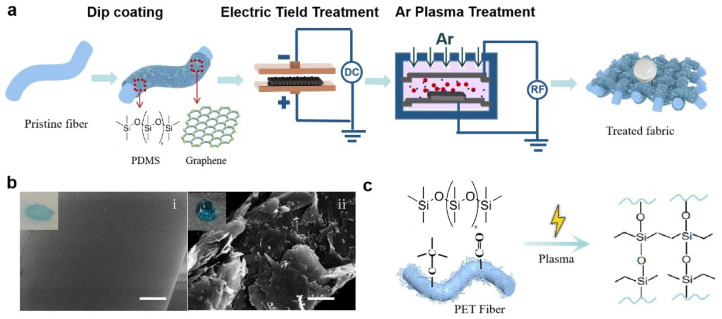
Preparation of superhydrophobic conductive fabrics. (**a**) Schematic diagram of the preparation of superhydrophobic conductive fabrics by electric field, Ar plasma treatment. (**b**) Scanning electron microscope images show the surface morphology of the fibers before and after treatment, with the inset showing the state of the dyed water droplets on the fabric surface. (**i**) is untreated fabric, (**ii**) is the fabric after PDMS/graphene wrapping. Scale bar: 2.5 μm. (**c**) Schematic diagram of the reaction principle between PDMS and polyester fibers in the plasma reaction chamber.

Figure 1a shows the fabrication process of the superhydrophobic conductive fabric. The preparation of the fabrics involves three steps: the dipping–coating process of graphene/PDMS precursor, the drying directional curing in the electric field, and the radio frequency capacitively coupled Ar plasma (RF-CCP) treatment. As shown in Figure 1b, in the graphene dispersion, a layer of graphene nanosheets adhered firmly and uniformly to the surface. Compared with the modified sample, the surface of the original knitted sample is relatively smooth. Under Ar plasma exposure, ions penetrated the polymer, initiating the self-diffusion of polymer chains and subsequent cross-linking (Figure 1c). The bombardment produced broken bonds and small molecules and formed active sites on the polyester surface. In addition, in the cavity, repolymerization and cross-linking reactions occur between the polyester fabric and PDMS and some small molecules, after which a three-dimensional network interpenetrating structure is formed on the polyester surface. This may be due to the partial activation of -Si-CH_3_ as -Si-CH_2_- under high-energy plasma and subsequent polymerization into -Si-CH_2_-CH_2_-Si- [20,21,22,23]. The plasma discharge time has an effect on the hydrophobicity of the fabric. A discharge time of 20 s predicted the best hydrophobic performance (Figure S3). Rough surfaces and long alkyl chains are formed under the action of plasma. Due to the formation of these structures, the stability of the coating is enhanced and a hydrophobic effect is achieved. On one hand, the hydrophobicity of the coating protects it from liquid erosion during underwater sensing and increases its stability and durability. On the other hand, the formed coating has a certain encapsulation effect on graphene and reduces graphene shedding during service.

**Figure 2:**
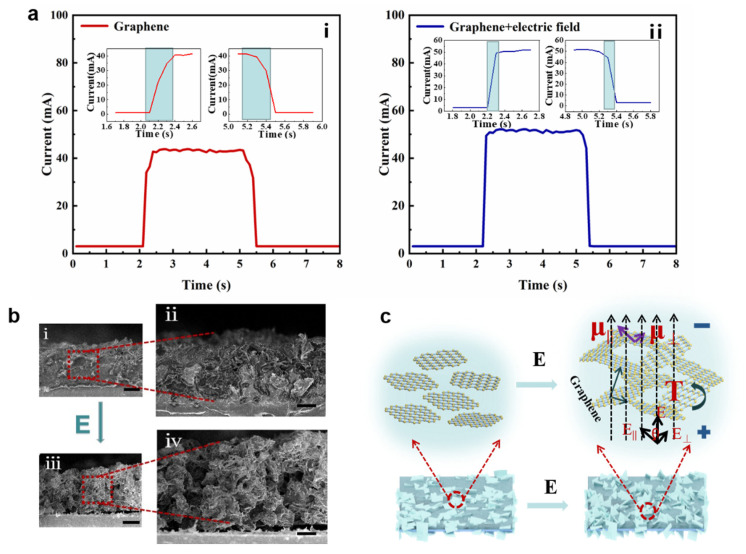
(**a**) Response/recovery time of the sensor at 50 kPa before and after electric field induction. (**a**(**i**)) is the response and response time of the unapplied electric field, (**a**(**ii**)) is the response and response time of the applied electric field. (**b**) The cross-sectional morphology of the coating before and after the electric field induction: (**b**(**i**),**b**(**ii**)) are the cross-sectional morphology before the electric field induction; (**b**(**iii**),**b**(**iv**)) are the cross-sectional morphology of the coating after the electric field induction. (**b**(**i**),**b**(**iii**)) scale bar: 50 μm; (**b**(**ii**),**b**(**iv**)) scale bar: 25 μm. (**c**) Schematic diagram of graphene nanosheets being twisted by force in an electric field.

An electric field is applied to graphite nanosheets on PDMS substrates induce directional alignment of nanosheets. To clarify the effect of the electric field on the sensitivity of the fabric sensor, the response and recovery time of the sensor are measured when pressure is applied and removed from the sensor. As shown in Table 1, the preparation methods and materials used in our research and reported work are summarized, as well as the response performance of the obtained devices. We used a simple impregnation method combined with an electric field to obtain a device with short response and recovery times. As shown in Figure 2a, when 50 kPa pressure (the weight is a cylinder with a base radius of 0.5 cm and a height of 3 cm.) is placed on the sensor surface, the response and recovery times of the fabric sensor without the electric field are both 200 ms (Figure 2a(i)). In contrast, in Figure 2a(ii), after the electric field is applied, the response and recovery times of the fabric sensor are reduced to 100 ms. This phenomenon is explained by the scanning electron microscopy of the coating section (Figure 2b). To better observe the cross-sectional morphology of the coating, it was fixed on the PET film. The action of the electric field makes graphene sheets deflected along the direction of the electric field. This arrangement enables the lap of graphene sheets. PDMS can be used as a spatial support material to form a more three-dimensional spatial structure. The additional structural deformation can produce a faster response when external forces are applied. Therefore, response time and recovery time are reduced.

However, the graphene nanosheets are not twisted completely parallel to the electric field, which may be due to the joint influence of the viscosity of macromolecules and the force between graphene nanosheets, and the movement of graphene nanosheets is greatly hindered by space. Therefore, the graphene nanosheets tend to move in one direction as a whole. Figure 2c shows a schematic diagram of the electric field-induced preparation of directionally aligned graphene subjected to forces. The different dielectric properties and conductivity between graphene nanosheets and macromolecular PDMS lead to the induced dipole moment (*μ*). Here the dipole moment can be divided into two directions—the component parallel to the graphene sheet *μ_||_* and the component perpendicular to the graphene sheet *μ_⊥_*—and the force acting on the graphene sheet is the superposition of the two effects [27].

The interaction between the electric field force and dipole moment produces an electric field-induced torque *T* = *μ* × *E*, which is the driving force for the electric field to align the graphene, and the torque will try to twist the graphene, so that the graphene is aligned parallel to the electric field. However, due to the large aspect ratio of graphene, the generated dipole moment usually differs from the direction of the applied electric field force. The overall force acting on a graphene sheet is the superposition of moments caused by fields parallel and perpendicular to its axis; note that *E_||_* = *E* × cos*θ*; *E_⊥_*= *E* × sin*θ*, and *θ* is the angle between the direction of the DC electric field and sheet axis.

When *θ* satisfies certain conditions, the total force can deflect the graphene sheet against the resistance in the system. If the directional arrangement of graphene is achieved under an electric field, the main consideration is the relationship between the torque and resistance of the system, and graphene starts to deflect when the torque generated under the electric field exceeds the sum of the resistance of the graphene in the system. Therefore, the choice of electric field strength is an important starting point for achieving the directional deflection of graphene.

**Figure 3:**
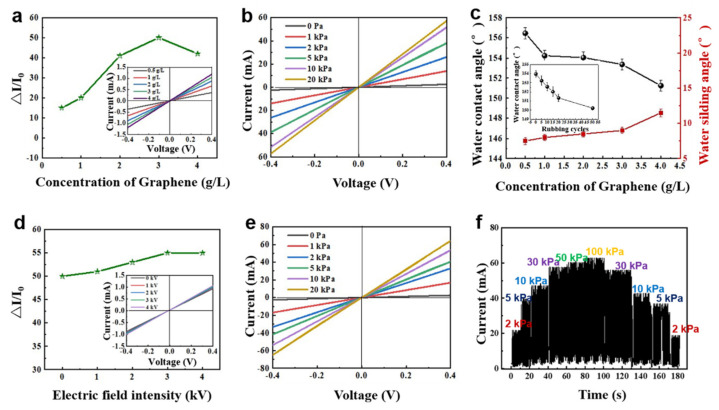
(**a**) ΔI/I_0_ value of the flexible pressure sensor at 50 kPa; the inset is the I−V curve of the single-layer fabric at different graphene concentrations. (**b**) I−V curves of the flexible pressure sensor under different static pressures. (**c**) Static and dynamic contact angles of the fabric after plasma treatment under different graphene concentrations; the inset shows the contact angle of the fabric after 50 rubbing cycles. (**d**) ΔI/I_0_ value of the flexible pressure sensor at 50 kPa after electric field induction; the inset is the I−V curve of the single-layer fabric under different electric field intensities. (**e**) I−V curves of the flexible pressure sensor under different static pressures after electric field induction. (**f**) Cycling test of the sensor under dynamic pressure after electric field induction.

The physical properties of the sensors are important for their performance. Therefore, the superhydrophobicity, electrical conductivity, and wear resistance of the sensors are discussed. Pressure sensors were assembled with graphene@PDMS samples with different graphene loadings and measured by an electrochemical workstation. As shown in the inset of Figure 3a, the changes in I−V curves of the single-layer fabrics with different graphene loadings are linear and pass through the origin, following Ohm’s law. The relative change in current (ΔI/I_0_, ΔI = I − I_0_, where I is the instantaneous current, I_0_ is the initial current at 0 kPa) is defined as the signal response of the pressure sensor, which can indirectly represent the sensitivity of the pressure sensor. The ΔI/I_0_ values initially increased and reaches an equal value then decreased as the dispersion concentration increased (Figure 3a). As a conductive material, graphene sheets lap each other and build a conductive network, which can reduce the resistance of composite fabrics effectively. However, when the additional amount of graphene is low, the distance between graphene sheets in the fabric is large, and they do not lap well with each other to form a complete conductive path. Thus low sensitivity and high resistance are caused. In contrast, the excessive amount of conductive material covering the fabric’s three-dimensional (3D) structure prevents the pressure sensor from producing large changes in contact resistance under pressure. This is because the conductive pathways have reached saturation and the number of conductive pathways no longer increases when external forces are applied [26]. The sensing performance test results of the sensor with different graphene contents at 30 kPa pressure showed that 3 g/L was selected as the best dispersion concentration for subsequent sensing performance tests. As shown in Figure 3b, the I−V curves of the graphene fabric pressure sensors showed a linear relationship under dynamic pressure. The resistance (obtained from the slope) is stable under different external pressures, indicating that the electrochemical performance of the sensor obeys Ohm’s law and has good piezoresistive performance. This has great potential for detecting changes in human surface pressure. In addition, the hydrophobicity of the fabric was tested. The results show that when the graphene concentration is 3 g/L, the interpenetrating cross-linking reaction between PDMS and polyester remained unaffected, and the coating can reach a superhydrophobic state. The static contact angle reached 153.96°, and the dynamic contact angle reached 9° (Figure 3c). The mechanical stability of superhydrophobic surfaces is also important in practical applications. This is because sensors inevitably rub against each other and other devices during service. The contact angle was measured to evaluate the mechanical durability of the sensor after different numbers of abrasion cycles. As illustrated in Figure 3c, the contact angle decreased from 153.9° to 149.3° after 50 abrasion cycles (Clamp the fabric in the radial weave direction to the standard wool tack cloth for experiments. Fix the white standard friction cotton backing fabric on the grinding head. The running speed is 2 s per friction cycle. The dynamic range of friction on the specimen is 50 mm.). The degradation of superhydrophobicity is due to the weaker bonded part of the PDMS coating being broken or peeled off from the fabric surface during external friction or mechanical rubbing.

The electric field was then introduced into the fabric treatment process, and the impregnated fabric was immediately placed in a DC electric field for directional twisting of graphene. As shown in the inset of Figure 3d, the I−V curves of the single-layer fabrics cured at different electric field strengths are all linear and through the origin, following Ohm’s law. The monolayer fabric still has a stable electrochemical output after being exposed to different voltages. From the slope of the I−V curves, the resistance of the fabric did not change significantly, indicating that the electric field had no obvious effect on the electrical conductivity of graphene nanosheets. As shown in Figure 3a,d, as the electric field was induced, the sensitivity ΔI/I_0_ of the sensor increased to approximately 55 and tended to be stable. This mainly depends on the deformation of the coating geometry. After orientation, the graphene coating has a fluffy and enlarged structure [18], and the adjacent graphene nanosheets generate tiny distances in space. The voids are mainly filled and supported by the PDMS elastomer (Figure 2b). Under the action of external force, the fluffy structure is deformed by force contraction, the nanosheets are in closer contact, the conductive paths are increased, and the ΔI/I_0_ value increases. However, after the voltage reaches 3 kV, the sensitivity of the sensor remains unchanged. This may be because torque and system resistance mainly affect graphene deflection in the electric field [27], indicating that the choice of voltage is an important starting point for realizing the torsion of graphene nanosheets. Conversely, we also applied a transverse electric field parallel to the coating direction. As illustrated in Figure S4, the response effect of the sensor decreases. We believe it is because the addition of the transverse electric field makes the stereospatial amplification structure disappear and there is no additional structural deformation under the external force (Figure S5). We then conducted a dynamic pressure test on the sensor assembled by the fabric after the action of the electric field. The I−V curves of the pressure sensor are also linear, and the resistance is stable under different external pressures, indicating that the sensor, after the electric field is applied, followed Ohm’s law and has good piezoresistive properties (Figure 3e). The cyclic stability of the fabric sensor was also tested after the electric field was applied. Figure 3f shows the sensing response under different external pressures. Notably, the pressure sensor has good repeatability and stable sensing response over a wide pressure range (2−100 kPa). The sensor effectively detects when the pressure is loaded from low to high. Likewise, the sensor detects the change in pressure from high to low. As well, at the same pressure, the pressure peak detection shows only a small drop (Figure S6). This shows that the sensing has good repeatability. However, it can be seen that when the pressure loaded exceeds 50 kPa, the sensitivity of the device to the pressure response decreases as the pressure continues to increase. This may be due to the structural deformation of the fabric reaching a critical value.

**Figure 4:**
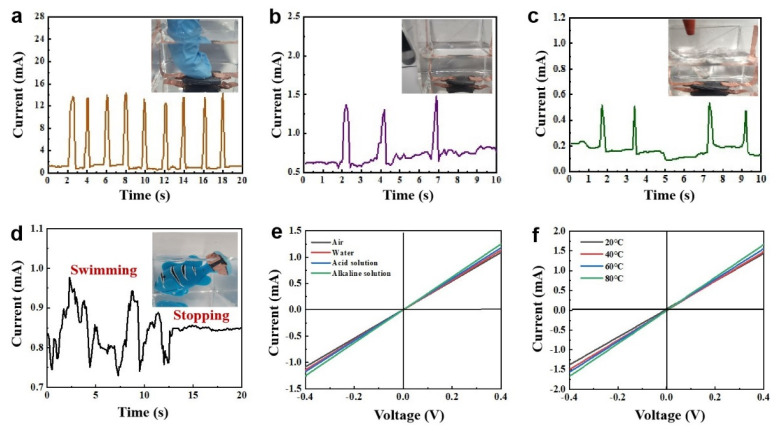
Resistive response and durability testing of sensors to different underwater behaviors. (**a**) Press the sensor in the water. (**b**) Tap the vessel wall with tweezers. (**c**) Blow on the water with an ear washing ball. (**d**) The resistance response curves of the small fish moving and stopping state; the inset is the simulated fish swimming mode to detect fish tail swing. (**e**) I−V response curves in different pH solutions. (**f**) I−V response curves of the sensor under different water temperature conditions.

To further discuss the practical use of the strain sensors, we also performed sensing tests on the superhydrophobic sensors in different underwater environments. This includes underwater crush detection, knockdown detection, and durability under extreme conditions [28]. As shown in Figure 4a, we pressed the sample in water, and the sensor produced a regular current response. This indicates that the conductive coating still has a stable output underwater. In addition, we conducted external vibration detection tests on the underwater sensors. As shown in Figure 4b, when the outer wall of the petri dish is tapped with tweezers, a regular resistance response can be observed, and the current response signal returns from peak to baseline level after each tap [12]. In addition, the sensor exhibits a slight change in resistance when the sample is placed on the bottom of the container with an ear-washing ball (Figure 4c). High sensitivity can extend the detection range of the sensor. For example, the sensor detects small pressure changes due to vocal cord vibrations (Figure S7). This may have implications for sensing vibrations and changes in liquid surfaces. Notably, the superhydrophobic sensor can also be used in underwater wearable devices. As shown in Figure 4d, we attached the fabric sensor to the fish to monitor the swimming and resting state of the small fish in real-time. During the swimming process of the simulation fish, we successfully detected resistance changes with the sensor. As shown in Figure 4e, we tested the electrochemical stability of the sensor in different underwater environments. The fabric sensor was placed on the bottom of the container, and solutions of different pH values were added to the container. The I−V curve of the sensor follows Ohm’s law under acidic, alkaline and neutral conditions. This indicates that the fabric still exhibits stable electrochemical properties under extreme conditions [29]. Finally, we placed the fabric sensor in water at different temperatures (Figure 4f), and the fabric exhibited good electrochemical performance; from the I−V curves, the resistance of the fabric increased as the temperature increased. The drop occurred in a small range, indicating that the fabric sensor may also be used to detect changes in underwater temperature.

## 4. Conclusions

In summary, we have demonstrated a fabric pressure sensor with high sensitivity, good stability and good superhydrophobicity using the electric field synergistic plasma approach. The fabric sensor exhibits higher sensitivity (ΔI/I_0_ of 55) and fast response time (~100 ms) due to the additional structural deformation obtained by the three-dimensional alignment of graphene under electric fields. The sensor can be used for pressure, water level, and underwater motion detection because it exhibits excellent superhydrophobicity (WCA = 153.9°) and good repeatability due to the formation of alkyl long chains and the rough surface under plasma. Moreover, stable superhydrophobic surfaces maintained good electrical conductivity when immersed in acid–alkali solutions with ultrasonic treatment. The sensor can be integrated into electronic circuits to provide warnings for preset pressures, or the fabric sensor could be used in underwater wearable devices to detect the pressure environment in which the human body is subjected to being underwater in real-time. We believe that this highly sensitive superhydrophobic fabric sensor provides an efficient strategy for wearable sensors, especially in wet or underwater environments.

## Figures and Tables

**Table 1:** Comparison between our work and related reported research.

Articles	Approaches	Materials	Response/Recovery Times
ours	Dipping/electric field	Graphene/PDMS	100 ms/100 ms
Huang et al. [24]	Spraying	MWCNTs/SEBS	/
Liu et al. [25]	brush coating/Spraying	CNF/PVDF/PDMS	/
Yu et al. [26]	Dipping	Ti_3_C_2_Tx MXene	300 ms/260 ms

## Data Availability

The data presented in this study are available within the article.

## Supplementary Materials

The following supporting information can be downloaded at: https://www.mdpi.com/article/10.3390/ma16041567/s1, Figure S1: DC electric field device diagram; Figure S2: The radio frequency capacitively coupled Ar plasma (RF-CCP); Figure S3: Effect of plasma discharge time on contact angle and roll angle of fabric; Figure S4: Pressure response curves of fabric flexible pressure sensors under different electric field induction directions; Figure S5: Cross-sectional morphology of composites under different electric field induction directions: (a) no electric field applied; (b) longitudinal electric field; (c) transverse electric field; Figure S6: Hysteresis test results; Figure S7: Vocal cord vibration monitoring; Table S1: Pharmaceutical chemistry measurement; Table S2 The sensing test system.
