# Supplementary material for: Underwater Highly Pressure-Sensitive Fabric Based on Electric-Induced Alignment of Graphene
Source: Materials (Basel). 2023 Feb 13;16(4):1567. doi: 10.3390/ma16041567 (PMC9961353; doi:10.3390/ma16041567)
Supplement: Supplementary file 1 [file materials-16-01567-s001.zip › materials-2079560-supplementary.pdf]

Supporting information

Underwater Highly Pressure-Sensitive Fabric Based on Electric-Induced Alignment of Graphene

Peiru Zhang [1,2], Lili Gu [1], Weiwei Liu [3], Dengteng Ge [4], Lili Yang [2], Jianjun Shi [1] and Ying Guo [1,*]

[1] Department of Applied Physics, Member of Magnetic Confinement Fusion Research Center, Ministry of Education, College of Science, Donghua University, Shanghai 201620, China
[2] State Key Laboratory for Modification of Chemical Fibers and Polymer Materials, College of Materials Science and Engineering, Donghua University, Shanghai 201620, China
[3] China Construction Advanced Technology Research Institute, China Construction Third Engineering Bureau Group Co., Ltd., Wuhan 430075, China
[4] Institute of Functional Materials, Donghua University, Shanghai 201620, China
* Correspondence: guoying@dhu.edu.cn; Tel.: +86-180-1790-5808

This word includes:
Figure S1 to Figure S7
Table S1 to Table S2

(1) Chemometrics of graphene, anhydrous ethanol and PDMS

Table S1. Pharmaceutical chemistry measurement.

Materials	Measurement
Graphene	0.6 g
Anhydrous ethanol	200 mL
PDMS	6 g

The reduced graphene oxide or graphene oxide is also a good candidate. Here, we chosen graphene due to these two reasons: (1) Graphene has the best electrical conductivity than graphene oxide or reduced graphene oxide. (2) Another reduction process of reduced graphene oxide should be added if we choose graphene oxide.

(2) The sensing test system

Table S2. The sensing test system.

Sensing Test Systems	Instrument Model
electrochemical workstation	CHI660E
sensor	2.2 in manuscript
applied pressure device	lab-built

The test system consists of an electrochemical workstation (CHI660E), a sensor, and a lab-built applied pressure device. The constant output voltage of the electrochemical workstation was set to 0.5 V. And the applied pressure frequency was changed ac-cording to the experimental requirements. First, the assembled sensor is connected to the instrument. Pressure is applied to the fabric surface with an applied pressure device. The electrochemical workstation records real-time current changes to test the pressure response of the sensor. Second, for sensing tests underwater, the fabric sensor is first fixed to the bottom of the vessel. An electrochemical workstation is attached to the fabric using copper foil conductive adhesive. The real-time current response of the underwater sensor is detected by applying vibration to the outer wall of the vessel or by applying external weight to the water surface.

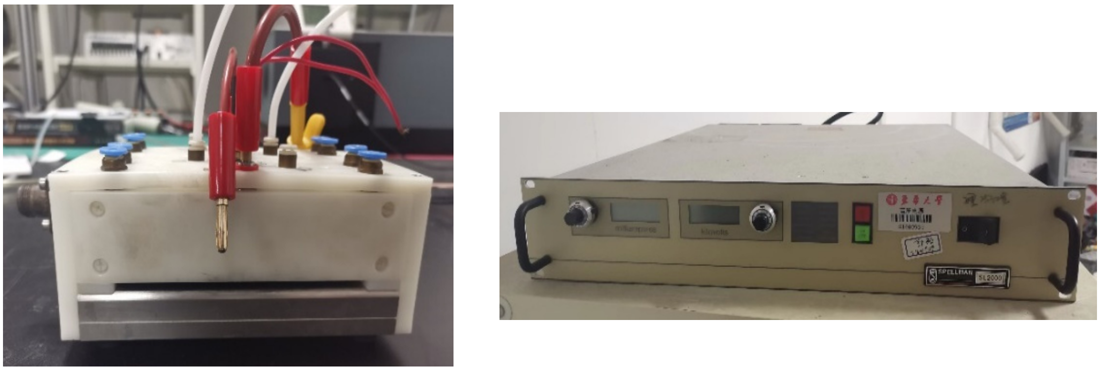

Figure S1. DC electric field device diagram.

The distance between the parallel plate electrodes was 0.5 cm.

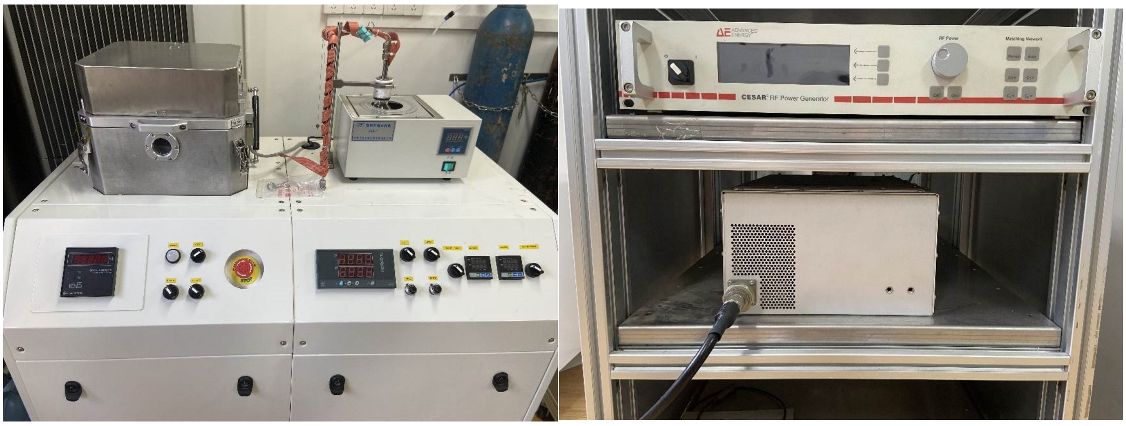

Figure S2. The radio frequency capacitively coupled Ar plasma (RF-CCP).

(3) The condition of plasma treatment and its advantages

The fabrics after the electric field action were plasma-treated in a radio frequency capacitively coupled plasma (RF-CCP) reactor (AP-600, North-March, USA) with the plasma discharge time of 20 s, discharge power of 50 W and discharge gas pressure of 50 Pa to obtain superhydrophobic conductive polyester fabrics.

There are many methods for the preparation of superhydrophobic surfaces. Plasma technology has been widely noted for its many advantages. For example, the technique does not produce toxic waste during the preparation process. No high temperature is required. Plasma also does not damage the original properties of the fabric. These advantages have led to the wide application of plasma technology in the field of textile material treatment.

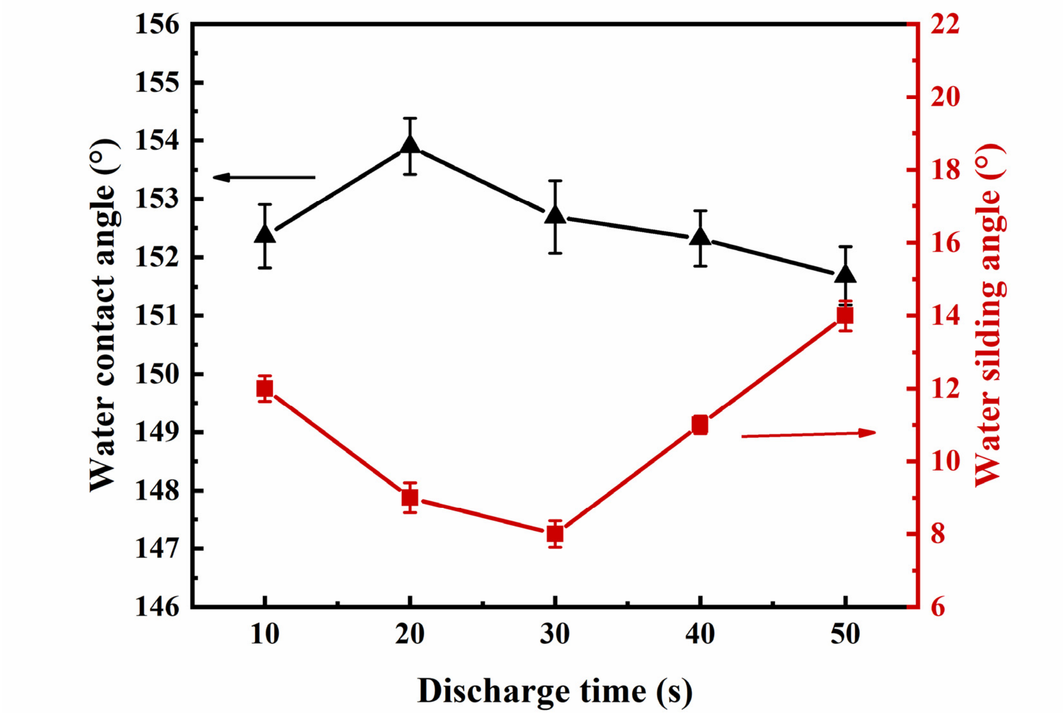

Figure S3. Effect of plasma discharge time on contact angle and roll angle of fabric.

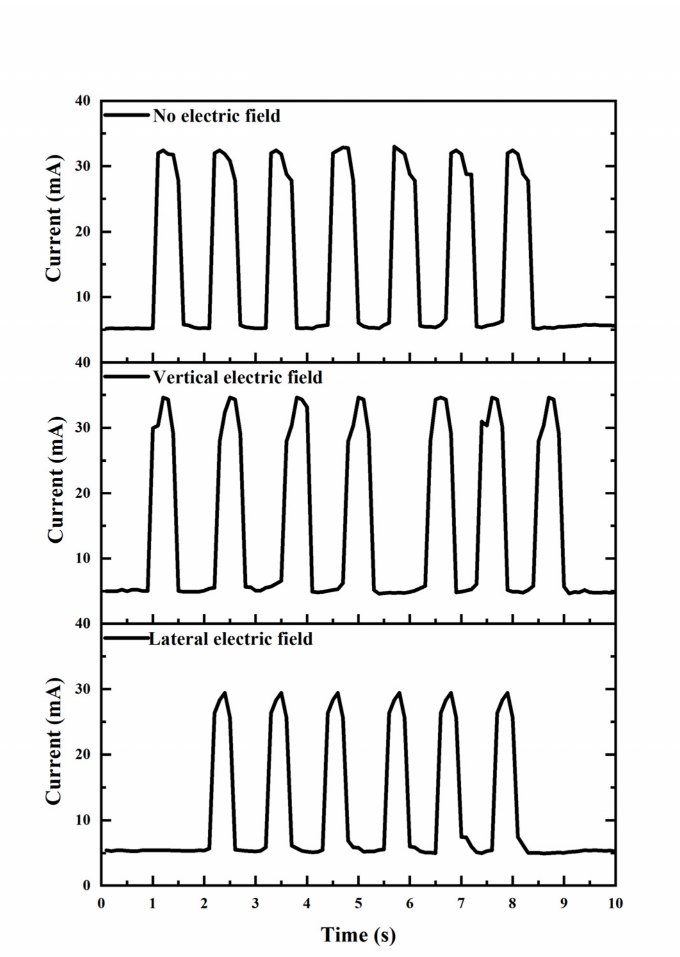

Figure S4. Pressure response curves of fabric flexible pressure sensors under different electric field induction directions.

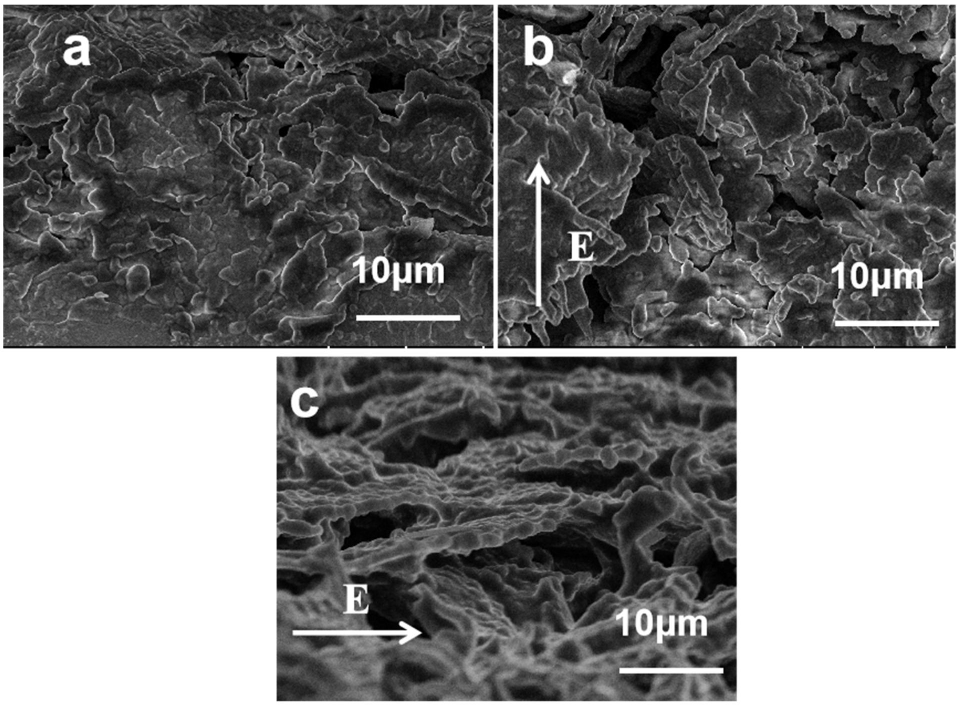

Figure S5. Cross-sectional morphology of composites under different electric field induction directions: (a) no electric field applied; (b) longitudinal electric field; (c) transverse electric field.

(4) Hysteresis test

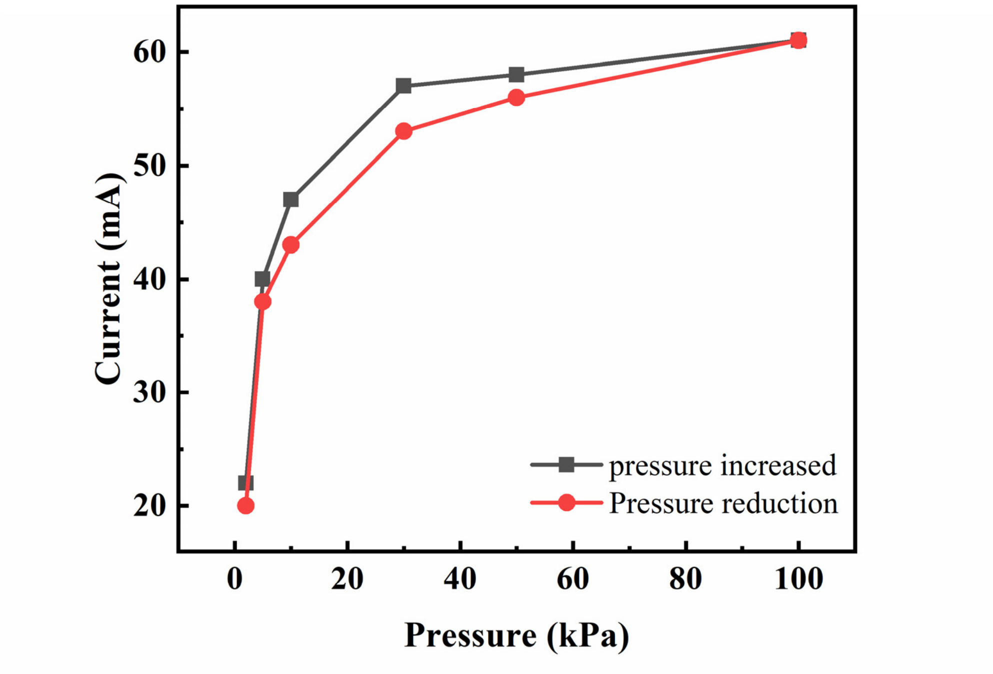

Figure S6. Hysteresis test results.

The main reason for the hysteresis is the interaction between graphene and PDMS between the mesh structure formed inside the PDMS. The graphene cannot return to its initial position quickly after the pressure is released.

As shown in Figure S6, the hysteresis is larger in low pressure. The hysteresis is smaller when the pressure is high. The PDMS/graphene composite does not recover quickly when the load is started to be applied. This leads to a high hysteresis phenomenon. The PDMS/graphene flexible pressure sensor has little deformation at larger pressures. Therefore the hysteresis is not significant.

(5) Vocal cord vibration monitoring

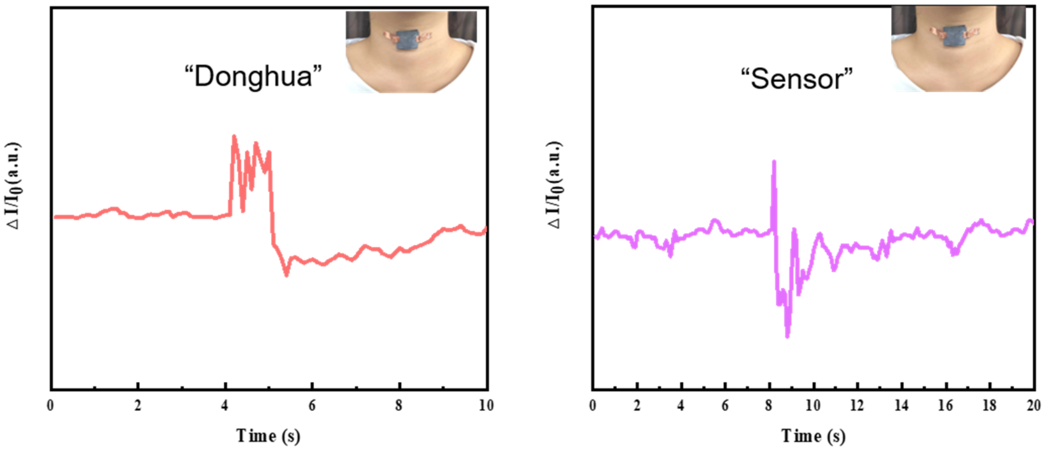

Figure S7. Vocal cord vibration monitoring.

In addition, we tested the sensor performance at 0.5～1 V operating voltage. The results show that 0.5 V can meet the test requirements. According to the reported work, the operating voltage range of the sensor focuses on between 0.5 V and 1 V. Therefore, 0.5 V is chosen as the performance test voltage of the sensor in this paper.
